# Morphine Reduces Expression of TRPV1 Receptors in the Amygdala but not in the Hippocampus of Male Rats

**Published:** 2014-05

**Authors:** Elham Hakimizadeh, Mohammad Kazemi Arababadi, Ali Shamsizadeh, Mohammad Allahtavakoli, Mohammad Ebrahim Rezvani, Ali Roohbakhsh

**Affiliations:** 1 Physiology-Pharmacology Research Center, Rafsanjan University of Medical Sciences, Rafsanjan, Iran;; 2 Immunology of Infectious Diseases Research Center, Rafsanjan University of Medical Sciences, Rafsanjan, Iran;; 3 Department of Physiology, School of Medicine, Shahid Sadoughi University of Medical Sciences, Yazd, Iran;; 4 Pharmaceutical Research Center, School of Pharmacy, Mashhad University of Medical Sciences, Mashhad, Iran

**Keywords:** Vanilloid receptor subtype 1, CA1 region, Amygdala, Morphine, Rats

## Abstract

**Background: **Chronic use of opioids usually results in physical dependence. The underlying mechanisms for this dependence are still being evaluated. Transient receptor potential vanilloid type 1 (TRPV1) are important receptors of pain perception. Their role during opioid dependence has not been studied well. The aim of this study was to evaluate the effect of morphine-dependence on the expression of TRPV1 receptors in the amygdala and CA1 region of the hippocampus.

**Methods:** This study used four groups of rats. Two groups of rats (morphine and morphine+naloxone) received morphine based on the following protocol: 10 mg/kg (twice daily, 3 days) followed by 20, 30, 40 and 50 mg/kg (twice daily), respectively, for 4 consecutive days. Another group received vehicle (1 ml/kg) instead of morphine given using the same schedule. The morphine+naloxone group of rats additionally received naloxone (5 mg/kg) at the end of the protocol. The control group rats received no injections or intervention. The amygdala and CA1 regions of the morphine, saline-treated and intact animals were isolated and prepared for real-time PCR analysis.

**Results: **Administration of naloxone induced withdrawal signs in morphine-treated animals. The results showed a significant decrease in TRPV1 gene expression in the amygdala (P<0.05) but not the CA1 region of morphine dependent rats.

**Conclusion: **TRPV1 receptors may be involved in morphine-induced dependence.

## Introduction

Opioids are important drugs in the treatment of moderate to severe pain. However, chronic use of opioids results in the development of antinociceptive tolerance and physical dependence. Dependence is revealed by a complex withdrawal syndrome associating physical (or somatic) signs with an intensely aversive emotional state.^1^ Historically, adenylyl cyclase, potassium and calcium channels, and the transmitter release have been considered in both opioid-induced analgesia and in antinociceptive tolerance. Today, diverse systems and targets are further implicated in the development of opioid dependence. Transient receptor potential vanilloid type 1 (TRPV1) is a member of a large family of ligand-gated ion channels. It is activated by capsaicin, the pungent ingredient found in hot chili peppers, resiniferatoxin (RTX), noxious heat (>43°C), low pH^2^ and numerous mediators.^3^ These channels are expressed in many brain regions^4^ with the highest level of TRPV1-like immunostaining in the hippocampus and cortex.^5^ While most studies on TRPV1 receptors have been conducted at the level of the spinal cord and peripheral structures, few studies have focused on brain structures.

There is several evidence regarding the existence of a functional interaction between opioid and TRPV1 receptors. For example, Endres-Becker et al.^6^have reported morphine reduced capsaicin-induced thermal allodynia. Furthermore, Chen and pan found that blockade expression of TRPV1 in the dorsal root ganglion (DRG) increases the analgesic effects of opioids.^7^ TRPV1, therefore, seems to have an antagonist effect on opioids. On the other hand, it has been documented that excessive and chronic administration of opioids can lead to increased pain;^8^ knock out TRPV1 mice do not develop this pain increase.^9^ It may be concluded that TRPV1 channels also play an important role in increased pain following chronic administration of opioids. Co-localization of TRPV1 and mu-opioid receptors (MOR) in DRG^10^ and the decrease in opioid ligand affinity in the rat brain upon capsaicin treatments^11^ also suggest the existence of a functional interaction. Previous studies have shown the involvement of both dorsal hippocampus and amygdala in opioid-induced conditioned place preference (reward).^12^^,^^13^ These regions are also involved in opioid dependence^14^ and critically participate in aversive memories of withdrawal syndrome in acute morphine-dependent rats.^15^ It has been reported that lesions in the amygdaloid complex reduce opioid-induced analgesia.^16^

Based on the above evidence, the present study was designed to determine mRNA expression levels of TRPV1 receptors in the CA1 region of the hippocampus and amygdala of morphine-dependent rats.

## Materials and Methods

We used 40 adult male Wistar rats weighed 225-300 g. Rats were housed in standard Plexiglass cages with free access to food and water. The animal house temperature was maintained at 23±2.0°C with a 12:12 h light/dark cycle. Animal handling and experimental method were approved by the Ethical Committee of Rafsanjan University of Medical Sciences. All efforts were made to minimize the number of animals and their suffering.

*Morphine Dependence and Withdrawal Model*

The rats were randomly categorized into four groups of 10 rats;^10^ control, saline, morphine (Daroupakhsh, Iran) and morphine+naloxone. According to a study by Cao et al.,^17^ the following procedures with some modifications were performed in order to establish a chronic morphine dependence model. Two groups of rats (morphine and morphine+naloxone) received 10 mg/kg of morphine intraperitoneally twice daily for the first 3 days and then from days four to seven they received 20, 30, 40 and 50 mg/kg of morphine, respectively, twice daily. The saline treated group received sterile 0.9% saline (1 ml/kg) instead of morphine as the same protocol. For evaluation of morphine dependence, one group (morphine+naloxone) received 5 mg/kg naloxone (Daroupakhsh, Iran) intraperitoneally 2 h following the last dose of morphine. The animals were placed in a Plexiglas cage (25 cm in diameter, 40 cm height) and the withdrawal syndrome signs were recorded as described elsewhere.^18^ Withdrawal signs were measured for 10 min starting 5 min after the naloxone injection.

The rats in the control group did not receive any injection or intervention. One hour after the final injection of morphine or saline, all rats (including control) were decapitated. Their amygdala and CA1 regions were isolated and stored within cerebrospinal fluid at -70° C until real-time PCR analysis. 

*Sample Preparation, RNA Extraction, Reverse Transcription and Quantitative Real-Time PCR*

Total RNA was extracted using the RNX extraction kit (Cinnaclon Company, Iran) according to the manufacturer’s guidelines. The extracted RNA quality was determined by measuring absorption at 260/280 nm by a UV spectrophotometer and electrophoresis on an ethidium bromide pretreated agarose gel. The extracted RNA was converted to cDNA using a cDNA synthesis kit (Parstous, Iran) using both oligo(dT) and random hexamer primers (table 1). Real-time PCR analyses were performed in triplicate and a β_2_-microglobulin (β2m) control was used for normalization of the amplification signal of the target genes. The relative amounts of PCR product were determined by the 2^-DDCt^ formula. In order to confirm the results, PCR products were electrophoresed parallel with a 50 bp ladder on 1% gel agarose that contained 0.5 mg/ml ethidium bromide.

**Table 1:** Gene-specific primers for amplification of rat transient receptor potential vanilloid type 1 (TRPV1) and β_2_microglobulin (β_2_m) mRNAs by real-time PCR

	**TRPV1 (accession no. NM_031982)**	**β2m ** **(accession no. NM_012512)**
**Forward primer**	5′-ACTCCTGACGGCAAGGATGAC-3′	5′-CCTGGCTCACACACTGAATTCACAC-3′
**Reverse primer**	5′-ACCCACATTGGTGTTCCAGGTAG-3′	5′-AACCGGATCTGGAGTTAAACTGGTC-3′
**Estimated size**	81 bp	163 bp

*Data Analysis and Statistical Methods*

Statistical analysis was performed using ANOVA. Following a significant F-value, post-hoc analysis (Tukey’s test) was performed for assessing specific group comparisons. To compare withdrawal signs, statistical analysis was performed using the *t* test. P values less than 0.05 were considered significant.

## Results

*Naloxone Precipitated the Withdrawal Syndrome*

Administration of naloxone following the last dose of morphine precipitated a well-defined withdrawal syndrome that included escape jumps, wet dog shakes, rearing, body scratching, penile licking and head washing in the morphine+naloxone group which indicated morphine-induced physical dependence. The results are presented in table 2.

**Table 2:** Withdrawal signs in morphine-treated rats compared with control saline-treated rats

**Signs**	**Control**	**Morphine**
Jumping	1.5±0.42	6.37±0.62***
Rearing	8.75±1.31	18.75±1.72***
Penile licking	4±0.37	7.37±0.82*
Body scratching	7.75±0.61	12.75±0.92***
Head washing	5.75±0.81	9.87±0.98**
Percentage of weight loss	0.62±0.26	8.25±2.3**

*P<0.05; **P<0.01; ***P<0.001 compared with the control group.

*Effects of Morphine-Dependence on TRPV1 Gene Expression*

**Figure 1:**
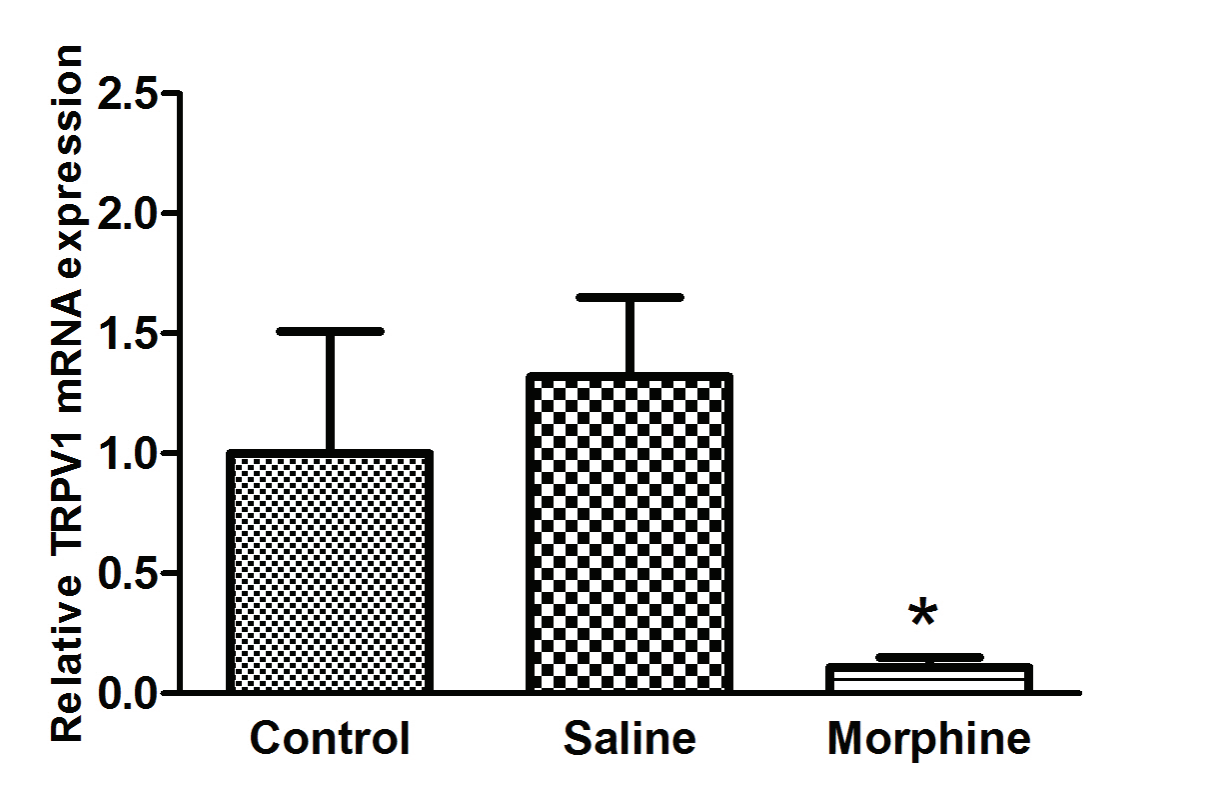
The effects of morphine dependence on mRNA expression level of transient receptor potential vanilloid type 1 (TRPV1) in the amygdala: *P<0.05 compared with the saline group. All data are presented as mean±SEM (n=10).

The results showed that mRNA expression levels of TRPV1 significantly decreased by 9.09 fold (P=0.013) in the amygdala of rats that received morphine compared to saline treated rats (figure 1).

**Figure 2:**
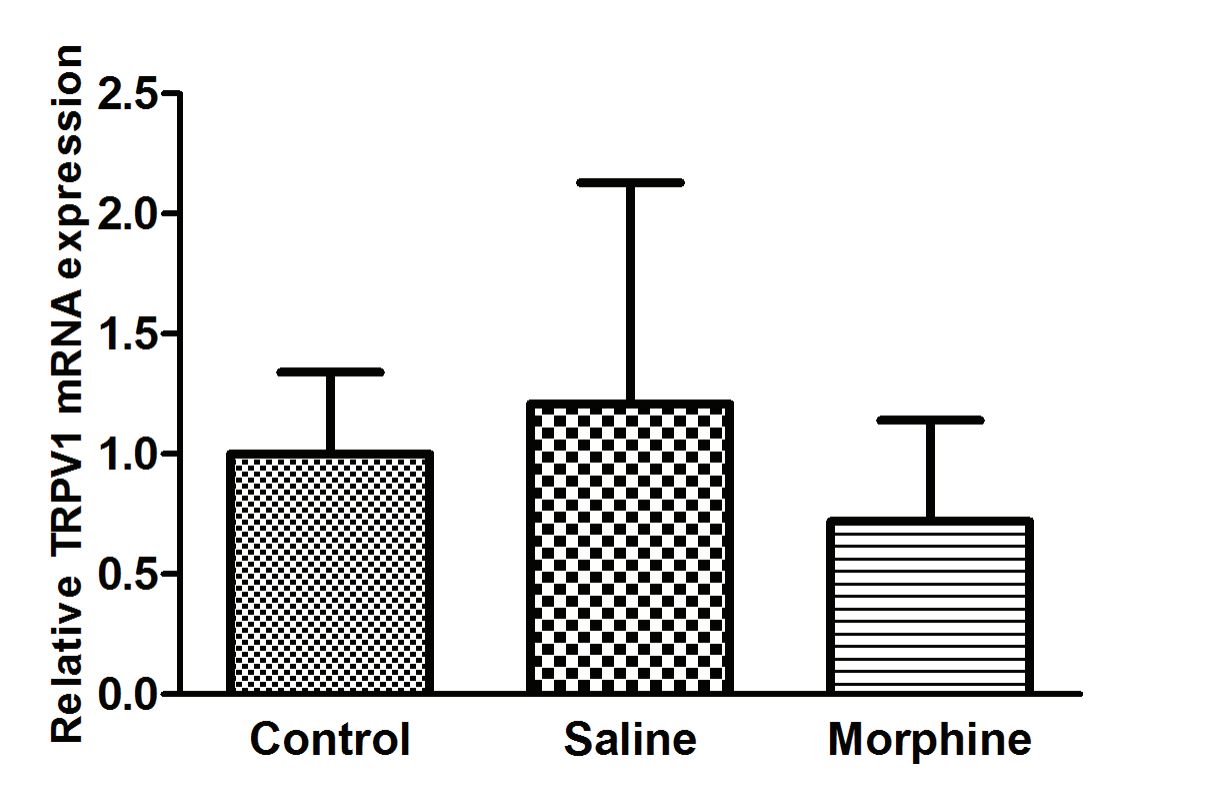
The effects of morphine dependence on mRNA expression level of the transient receptor potential vanilloid type 1 (TRPV1) gene in the CA1 region of the hippocampus. All data are presented as mean±SEM (n=10).

The results also revealed that TRPV1 mRNA expression levels in CA1 region of rats that received morphine injections did not change significantly compared with saline treated rats (P>0.05; figure 2).

## Discussion

This study was undertaken to evaluate the role of morphine dependence on mRNA levels of the TRPV1 receptor in the amygdala and hippocampus. 

Our findings demonstrated that following morphine administration, TRPV1 receptor mRNA levels reduced in the amygdala. Additionally, our results showed that TRPV1 mRNA expression in the CA1 structure did not change significantly compared with saline treated rats. The current finding also highlighted the important role of the amygdala in morphine dependence as has been reviewed previously^19^and showed that the effects of morphine on TRPV1 receptors is target dependent. Considering the important role of the amygdala in morphine antinociception,^20^ it may be suggested that a gradual decrease in TRPV1 receptor expression in the amygdala but not in the hippocampus is also involved in the antinociception effect of morphine. The different effect of morphine on TRPV1 mRNA level in the amygdala and hippocampus may also be explained by the different role of these regions in modulating anxiety. Regarding the distinct role of the amygdala in anxiety-like behaviors^21^ and the anxiogenic effect of TRPV1 receptors,^22^^,^^23^ it may be suggested that reduced TRPV1 mRNA in the amygdala but not in the hippocampus partly mediates morphine-induced anxiolysis.^24^

In accordance, previous studies have shown the existence of a functional and complex interaction between opioid and TRPV1 receptors. For example, capsaicin-induced thermal allodynia is attenuated by stimulating MOR opioid receptors in the central nervous system of rhesus monkeys.^25^ On the other hand, it has been reported that SB366791 and capsazepine as TRPV1 receptor antagonists suppress analgesic tolerance and physical dependence to morphine^10^^,^^26^ and the development of tolerance to morphine is substantially attenuated in the absence of TRPV1-expressing primary afferent neurons of the RTX-treated rats.^27^ In a very recent study, Spahn and colleagues have demonstrated that TRPV1 activity increased in DRG neurons during morphine withdrawal syndrome.^28^Although the authors did not evaluate the role of central TRPV1 receptors, they have concluded that change in TRPV1 activity during opioid withdrawal syndrome is a new mechanism that contributes to opioid withdrawal-induced hyperalgesia. In contrast, it has been reported that capsaicin and the MOR receptor agonist, DAMGO, when co-administered into the ventrolateral-periaqueductal gray at non-analgesic doses per se induce analgesic effects^29^ and capsaicin can inhibit some morphine withdrawal symptoms in rats.^30^

The mechanism by which opioids affect TRPV1 receptors may be divided into rapid and delayed effects. Opioids via G_i/o _proteins in a cAMP/PKA-dependent pathway decrease translocation and multimerization of TRPV1 channels from an intracellular store of inactive TRPV1 monomers in the membranes of target cells.^31^ This effect has been suggested as a cellular mechanism for rapid and fine tuning of TRPV1 responses independent of transcriptional changes. This suggestion was further supported by the ability of opioids to inhibit capsaicin responses potentiated by cAMP-dependent PKA.^32^ The results of a study by Spahn has also shown that activation of TRPV1 receptors during opioid withdrawal is cAMP dependent and mediated through activation of protein kinase A.^28^Therefore, opioids by inhibition of protein kinase A rapidly modulate TRPV1 receptor activity. The delayed effects of opioids may be presented by changes in TRPV1 receptor transcription and/or translation. For example, it has been reported that TRPV1 mRNA increased in the spinal cord and sciatic nerve (2.7 and 3.9 fold, respectively) and decreased in the DRG of morphine-treated rats.^10^ This finding in conjunction with the results of the present study suggests that change in TRPV1 mRNA level during morphine dependence is target dependent.

Likewise, TRPV1 receptors may also change the expression of opioid receptors.^7^ The mechanism by which opioid and TRPV1 receptors induce reciprocal changes in expression has not yet been studied. Two recent studies show that GABA_A_ receptor associated protein (GABARAP) is involved in the expression of both TRPV1 receptors and opioid receptors.^33^^,^^34^ Thus, it may be suggested that this protein possibly mediates the interaction of opioid and TRPV1 ligands. The delayed effects of opioids on TRPV1 receptors may also be represented during opioid-induced hyperalgesia. Clinical studies have reported that opioids administered, particularly during rapid opioid dose escalation, can produce hyperalgesia and allodynia.^8^ Similarly, the study of Vardanyan et al.^9^ shows that unlike wild-type mice, TRPV1 knock-out mice do not develop thermal and tactile hypersensitivity induced by sustained morphine administration and morphine increases TRPV1 immunoreactivity in the DRG and induces functional changes in TRPV1 receptor at the periphery.

## Conclusion

It may be concluded that TRPV1 receptors have a role in opioid dependence. More studies are required to evaluate the interaction of TRPV1 and opioid receptors in detail. 
